# Correlation between Cholesterol, Triglycerides, Calculated, and Measured Lipoproteins: Whether Calculated Small Density Lipoprotein Fraction Predicts Cardiovascular Risks

**DOI:** 10.1155/2017/7967380

**Published:** 2017-11-28

**Authors:** Sikandar Hayat Khan, Nadeem Fazal, Athar Abbas Gilani Shah, Syed Mohsin Manzoor, Naveed Asif, Aamir Ijaz, Najmusaqib Khan Niazi, Muhammad Yasir

**Affiliations:** ^1^Department of Pathology, PNS Hafeez, Islamabad, Pakistan; ^2^Department of Medicine, PNS Hafeez, Islamabad, Pakistan; ^3^Department of Surgery, PNS Hafeez, Islamabad, Pakistan; ^4^Department of Chemical Pathology & Clinical Endocrinology, AFIP, Rawalpindi, Pakistan; ^5^Administration Department, PNS Hafeez, Islamabad, Pakistan

## Abstract

**Background:**

Recent literature in lipidology has identified LDL-fractions to be more atherogenic. In this regard, small density LDL-cholesterol (sdLDLc) has been considered to possess more atherogenicity than other LDL-fractions like large buoyant LDL-cholesterol (lbLDLc). Recently, Srisawasdi et al. have developed a method for calculating sdLDLc and lbLDLc based upon a regression equation. Using that in developing world may provide us with a valuable tool for ASCVD risk prediction.

**Objective:**

(1) To correlate directly measured and calculated lipid indices with insulin resistance, UACR, glycated hemoglobin, anthropometric indices, and blood pressure. (2) To evaluate these lipid parameters in subjects with or without metabolic syndrome, nephropathy, and hypertension and among various groups based upon glycated hemoglobin results.

**Design:**

Cross-sectional study*. Place and Duration of Study*. From Jan 2016 to 15 April 2017.

**Subjects and Methods:**

Finally enrolled subjects (male: 110, female: 122) were evaluated for differences in various lipid parameters, including measured LDL-cholesterol (mLDLc), HDLc and calculated LDL-cholesterol (cLDLc), non-HDLc, sdLDLC, lbLDLC, and their ratio among subjects with or without metabolic syndrome, nephropathy, glycation index, anthropometric indices, and hypertension.

**Results:**

Significant but weak correlation was mainly observed between anthropometric indices, insulin resistance, blood pressure, and nephropathy for non-HDLc, sdLDLc, and sdLDLc/lbLDLc. Generally lipid indices were higher among subjects with metabolic syndrome [{sdLDLc: 0.92 + 0.33 versus 0.70 + 0.29 (*p* < 0.001)}, {sdLDLc/lbLDLc: 0.55 + 0.51 versus 0.40 + 0.38 (*p* = 0.010)}, {non-HDLc: 3,63 + 0.60 versus 3.36 + 0.65 (*p* = 0.002)}]. The fact that the sdLDLc levels provided were insignificant in Kruskall Wallis Test indicated a sharp increase in subjects with HbA1c > 7.0%. Subjects having nephropathy (UACR > 2.4 mg/g) had higher concentration of non-HDLc levels in comparison to sdLDLc [{non-HDLc: 3.68 + 0.59 versus 3.36 + 0.43} (*p* = 0.007), {sdLDLc: 0.83 + 0.27 versus 0.75 + 0.35 (*p* = NS)}].

**Conclusion:**

Lipid markers including cLDLc and mLDLc are less associated with traditional ASCVD markers than non-HDLc, sdLDLc, and sdLDLc/lbLDLc in predicting metabolic syndrome, nephropathy, glycation status, and hypertension.

## 1. Introduction

Atherosclerotic cardiovascular diseases (ASCVD) have emerged as the leading cause of human morbidity and mortality across all races and ethnicities. Literature review strongly signifies the increasing frequency of stroke, IHD, peripheral vascular disease (PVD), and diabetes in subcontinental countries and countries with emerging economies [1]. In the developing world the concept of adipocytes having “thrifty genotype” and “starvation genes” has been associated with higher prevalence of diseases resulting from ASCVD [2].

Genetics, lifestyles, and environmental triggers can all help in accelerating cholesterol deposition to cause ASCVD. Traditionally the ultimate villain in this interplay had always been the (low density lipoprotein cholesterol) LDLc [3]. The convention to date had seen the plight of lipoproteins classification as good and evil, that is, HDLc and LDLc, with most literature guidelines relying upon them as diagnostic and clinical intervention markers in managing various categories of ASCVD [4, 5]. However, various evolving technologies have now allowed the researchers to measure and study the role of different subclasses of lipoproteins [6]. An insight into defining these lipoproteins is technically based upon their particular size, which vary from less than 1.06 (LDL) and greater than 1.06 nm to 1.23 nm as HDL after segregation through ultracentrifugation. [7]. These lipoproteins are actually mixtures of various proportions of esterified and nonesterified cholesterol, phospholipids, proteins, triglycerides, and surface apolipoproteins [8]. Kinetic studies have identified a lot of variability in terms of shape, size, and lipid composition which are difficult to measure as perfection in clinical laboratories provided improvement in laboratory science and calibration practices [9]. The recent data has subcategorized LDL particles based upon their size and density into small and dense LDL-cholesterol particles termed small density LDLc (sdLDLc) and large dense LDL-cholesterol, which has been proven to be more predictive to highlight underlying cardiovascular risks [10]. The former category of lipoproteins is now considered to easily penetrate vessel wall to become oxidized and thus causing nondesirable ASCVD outcomes [11]. Thus current evolution in lipidology is now converging to recognize the importance of sdLDLc in causation of ASCVD risks; however, the technologies measuring LDL particle number are yet not available in most developing healthcare markets along with cost-effectiveness being another consideration. Srisawasdi et al. have recommended a surrogate for measuring sdLDLc and lbLDLc by utilizing mathematical modeling incorporating step wise multivariate regression equation and recommended its use for worldwide clinical practice [12]. Koba et al. have also observed that LDL mass rather than size is more significant as LDL particle concentration in IHD progresses [10]. Moreover, the same authors have also felt that the risk predicting capability of sdLDLc is superior to that of non-HDL cholesterol and LDL-cholesterol.

With this background information the authors have decided to study the correlation of calculated small dense LDL-cholesterol (sdLDLc) and calculated large buoyant LDL-cholesterol (lbLDLc) and traditional lipid markers with varying ASCVD associated risk factors based upon glycemic status, insulin resistance (IR) status, nephropathy status, metabolic syndrome, and blood pressure.

## 2. Materials and Methods

After formal approval by hospital's ethical review committee, this cross-sectional study was conducted at department of pathology and medicine, PNS Hafeez (Islamabad), and department of chemical pathology and endocrinology, Armed Forces Institute of Pathology (AFIP), Rawalpindi. The study duration was 1 year starting from Jan 2016 to Jan 2017. From a target population of referrals from medical and surgical OPD subjects to laboratory for estimation of lipid profile and fasting plasma glucose, 232 OPD subjects were finally enrolled after complete explanation of study concept, probable outcomes, and nature of clinical interventions involved with formally signing the consent form. Subjects who had some chronic or acute disorder, pregnancy, children, and admitted cases on medication known to alter lipid/related parameters were excluded from the study. Few samples were excluded later due to hemolysis and related technical reasons. The OPD patients were interviewed according to predesigned clinical Performa and were clinically evaluated using various anthropometric indices as per WHO criteria [13]. 10 ml of blood was drawn in EDTA, plain bottles, and Na-Fluoride tubes for measuring various biochemical parameters. Fasting plasma glucose, cholesterol, and triglycerides were measured using GOD-PAP, CHOD-PAP, and GPO-PAP method on Selectra-ProM, while (measured LDLc) mLDLc and HDLc were measured by cholesterol esterase method on ADVIA 1800 Chemistry System, respectively. Calculated LDLc (cLDLc) was measured using Friedewald's formula and sdLDLc and lbLDLc were calculated as per the regression equation recommended by Srisawasdi et al. [12] as follows:

(1) sdLDL-c mmol/L=0.580non-HDL-c+0.407mLDL-c−0.719cLDL-c−0.312.

Glycated hemoglobin was measured using fast ion-exchange resin separation method; serum insulin by chemiluminescence's technique on Immulite® 1000 and spot urine specimen in 174 subjects for measuring urine albumin creatinine ratio (UACR) were evaluated by immunoturbidimetric method on ADVIA 1800. Homeostasis Model Assessment for insulin resistance (HOMA-IR) was calculated as per the method of Matthews' et al. [14]. Metabolic syndrome was diagnosed using (National Cholesterol Education Program) NCEP and International Diabetic Federation (IDF) criteria [15, 16]. Based upon glycated hemoglobin results, four groups were made, namely, Group-1: HbA1c levels < 5.5%, Group-2: HbA1c levels = 5.6–6.5%, Group-3: HbA1c levels = 6.6–7.0%, and Group-4: HbA1c levels > 7.0%. Two groups for nephropathy related impact were made based upon patient's UACR results as Group-1 with UACR < 2.5 mg/g and Group-2 with UACR > 2.4 mg/g.

### 2.1. Data Analysis

All data were entered into Excel program (Microsoft Office-2007) and later transferred into SPSS version-15. Descriptive statistics in terms of mean ± SD were calculated for age. All lipid indices were compared between gender groups through independent sample *t*-statistics. Pearson's correlation was calculated between various lipid parameters with anthropometric indices, blood pressure, and biochemical risk factors. Nonparametric “Kruskal Wallis Test” was employed to compare various groups formulated based upon the presence or absence of metabolic syndrome components (as per the IDF criteria) to compare lipid parameters and later the same test was employed to compare various groups formulated upon the glycated hemoglobin results for the ratio between small density and large buoyant LDL-cholesterol. Independent sample *t*-test was employed to compare lipid indices between subjects with or without metabolic syndrome and subjects with or without nephropathy based upon UACR results. Hypertensive and nonhypertensive groups were compared for various lipid indices by employing Mann–Whitney *U* test.

## 3. Results

**Figure 1:**
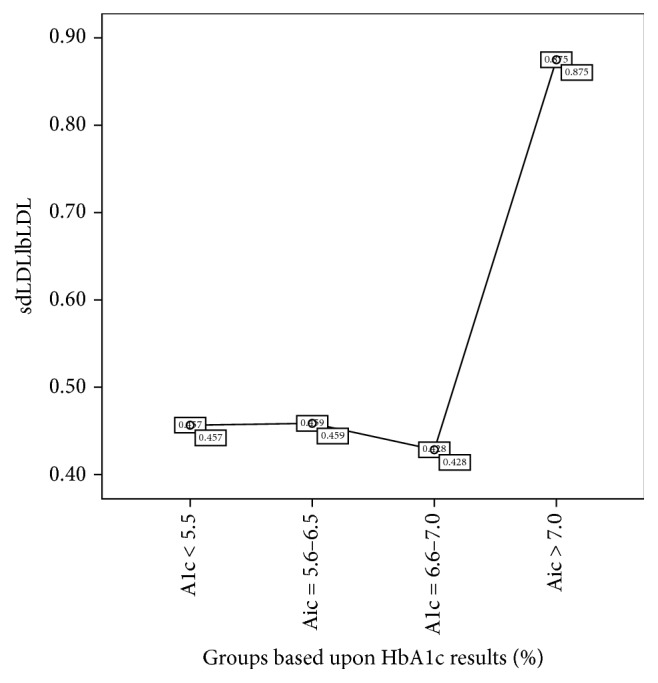
Comparison between groups based upon HbA1c values for sdLDLc/lbLDLc by Kruskal Wallis Test (*p* = 0.430).

The study population constituted 122 females with age 45.27 + 12.42 years and 110 males with 47.98 + 11.30 years. Gender-wise comparison for various lipid parameters is depicted in Table 1 where differences were significant for HDLc, non-HDLc, and LDLc.  Table 2 demonstrates Pearson's correlation for lipid parameter with anthropometric, blood pressure, and biochemical risk factors, where non-HDLc, sdLDLc, and sdLDLc/lbLDLc were found to be better correlated with aforementioned designated risk factors. The differences for non-HDLc and sdLDLc were found to be most significant among subjects with or without metabolic syndrome (Table 3). Assessing metabolic cluster-wise increment (as per metabolic syndrome definition) we observed that (excluding criteria inclusive markers like triglycerides and HDLc), serum non-HDLc, sdLDLc, and sdLDLc/lbLDLc increased gradually among subjects with no component to subjects having all components of metabolic syndrome (Table 4). The results for various glycated hemoglobin based groups for sdLDLc/lbLDLc were not found to be significant which may be due to noninclusion of known diabetics. However, Figure 1 suggests a rapid increase in the number of sdLDLc in comparison to lbLDLc (sdLDLc/lbLDLc) with patient HbA1c group having HbA1c > 7.0%; however, the results were not significant but authors feel that type-2 statistical error due to small size of group-4 (*n* = 12) could be one reason behind this nonsignificance. There were no differences among any of the lipid markers between subjects with or without hypertension (Table 5). Based upon urine albumin creatinine ratio (UACR) we only observed significant differences for non-HDLc and cLDLc (Table 6).

## 4. Discussion

Calculated sdLDLc and its ratio with lbLDLc have provided marginally improved risk prediction by being better and significantly correlated with multiple traditional and established ASCVD markers. In this regard it is important to appreciate that sdLDLc levels were clearly found to be increased in subjects having metabolic syndrome and insulin resistance and these levels increase in a staircase manner from no risk factors to acquiring all five components of metabolic syndrome as also demonstrated by other researchers [17, 18]. However, it appears that other lipid markers especially non-HDLc, VLDLc, triglycerides, and HDLc also worsened with accumulation of various metabolic cluster which brings us to the reality that these lipoprotein bound and free lipids are constantly modifying and contributing to each other. Therefore the previously used entity of “atherogenic dyslipidemia” being low HDLc and high triglycerides can be broadened to also include increases in sdLDLc, non-HDLc, and VLDLc [18–20].

Non-HDLc showed more correlation with BMI and WhpR than other lipid markers including sdLDLc and its ratio with lbLDLc; however, the latter seem to be better associated with WhpR. Recent studies have also highlighted WhpR to be more predictive of ASCVD risk than BMI which seems to be more representative of muscle mass [21, 22].

Glycation rates have been associated with enhanced atherosclerosis and morbidity and mortality liked to CVD [23]. In this regard our study which did not include any known diabetics has only demonstrated sdLDLc/lbLDLc ratios to have mild weak correlation with glycated hemoglobin and slightly higher results group of diagnosed diabetics. This strengthens our viewpoint that some degree of lipid derangements does start with increasing glycation in the shape of increased numbers of small-sized LDL in comparison to large LDL particles in the plasma as highlighted by some researchers [23–26].

While both diastolic and systolic blood pressure are included in metabolic syndrome, still we could not observe significant differences for various lipid markers among hypertensive and nonhypertensive patients which is in line with the findings of Esteghamati et al. [27]. However, we found the ratio between sdLDLc/lbLDLc to have weak correlation with systolic and diastolic blood pressures, which indicates that slight derangements in lipid metabolism do develop in subjects having raised blood pressures [28, 29]. sdLDL/lbLDLc along with non-HDLc and mLDLc/HDLc did show some weak correlation with UACR but it was only non-HDLc that demonstrated significant differences between subjects with and without nephropathy. These findings are consistent with the results of Palazhy et al. [30, 31].

Certain* limitations* to the study must be acknowledged. We have utilized Srisawasdi et al.'s regression equation for measuring sdLDLc and lbLDLc, which still needs to be validated by epidemiological studies. Moreover, our study has small sample size and cross-sectional design where type-2 statistical errors could have confounded our findings so large clinical randomized clinical trials may be carried out to augment or disapprove our observations.

The is a* clinically important* study as it not only has highlighted association between lipid parameters with various traditional risk factors but also has allowed us to understand how different lipid indices vary across various anthropometric and biochemical groups. The study has also opened up some new avenues for research on LDL-fractions so as to learn in detail the risk association between lipoprotein indices and cardiovascular diseases. Moreover, the study was also able to highlight the superiority of non-HDLc over available lipid indices in measuring ASCVD risk.

## 5. Conclusion

Calculated sdLDLc and its ratio with lbLDLc were not able to augment any ASCVD risk prediction over and above non-HDLc. However, it becomes apparent that other lipid markers including calculated LDLc and measured LDLc are less associated with traditional ASCVD markers than non-HDLc, sdLDLc, and sdLDLc/lbLDLc in predicting metabolic syndrome, nephropathy, glycation status, and hypertension. However, the results need to be validated by methods which directly measure sdLDLc or LDL-fractions.

## Figures and Tables

**Table 1:** Gender-wise comparison of various lipid indices.

Parameter	Gender	*N*	Mean	Std. deviation	Sig. (2-tailed)^*∗*^
Total cholesterol (mmol/L)	Male	110	4.54	0.59	0.171
	Female	122	4.43	0.62	
Fasting triglycerides (mmol/L)	Male	110	1.69	0.82	0.112
	Female	122	1.53	0.67	
*HDLc (mmol/L)*	*Male*	*109*	*0.91*	*0.21*	*0.000*
	*Female*	*121*	*1.04*	*0.28*	
mLDLc (mmol/L)^*∗∗*^	Male	108	2.71	0.68	0.583
	Female	122	2.66	0.76	
*Non-HDLc (mmol/L)*	*Male*	*110*	*3.63*	*0.58*	*0.008*
	*Female*	*122*	*3.41*	*0.68*	
*cLDLc (mmol/L)* ^*∗∗∗*^	*Male*	*110*	*2.88*	*0.51*	0.017
	*Female*	*122*	*2.70*	*0.57*	
sdLDLc (mmol/L)^*∗∗∗∗*^	Male	110	0.82	0.35	0.676
	Female	122	0.80	0.35	
lbLDLc (mmol/L)^*∗∗∗∗∗*^	Male	110	1.84	0.56	0.818
	Female	122	1.86	0.50	
sdLDLc/lbLDLc	Male	110	0.50	0.55	0.486
	Female	122	0.45	0.37	
*LDL-c/HDLc*	*Male*	*109*	*3.08*	*0.94*	*0.002*
	*Female*	*121*	*2.70*	*0.91*	
VLDL-cholesterol (mmol/L)	Male	110	0.34	0.16	0.112
	Female	122	0.31	0.13	

^**∗**^Measured using independent sample *t*-test (SPSS); ^*∗∗*^measured LDL-cholesterol (mLDLc) by cholesterol esterase method; ^**∗∗∗**^calculated LDL-cholesterol (cLDLc) by Friedewald's formula; ^**∗∗∗∗**^small density LDL-cholesterol (sdLDLc) by Srisawasdi et al. regression equation; ^**∗∗∗∗∗**^large buoyant LDL-cholesterol (lbLDLc) by Srisawasdi et al. regression equation.

**Table 2:** Correlation between various lipid parameters with anthropometric, blood pressure, and biochemical risk factors with lipid parameters.

	Total cholesterol	Fasting triglyceride	HDLc	mLDLc	Non-HDLc	cLDLc	sdLDLc	lbLDLc	sdLDLc/lbLDLc	mLDLc/HDLc
Body Mass Index (BMI)										
Pearson Correlation	0.197^*∗∗*^	0.115	0.126	0.032	*0.139* ^*∗*^	0.080	0.099	0.018	0.093	−0.045
Sig. (2-tailed)	0.003	0.081	0.056	0.626	*0.035*	0.224	0.132	0.783	0.160	0.500
*N*	232	232	230	230	*232*	232	232	232	232	230
Waist to hip ratio (WhpR)										
Pearson Correlation	0.205^*∗∗*^	0.173^*∗∗*^	−0.004	0.169^**∗**^	*0.191* ^*∗∗*^	0.123	*0.202* ^*∗∗*^	0.079	*0.122*	0.095
Sig. (2-tailed)	0.002	0.008	0.957	0.010	*0.004*	0.062	*0.002*	0.231	*0.063*	0.150
*N*	232	232	230	230	*232*	232	*232*	232	*232*	230
Glycated hemoglobin (HbA1c %)										
Pearson Correlation	−0.050	0.101	0.032	−0.011	−0.040	−0.132^*∗*^	0.074	−0.068	*0.149* ^*∗*^	−0.028
Sig. (2-tailed)	0.456	0.129	0.632	0.864	0.546	0.046	0.268	0.309	*0.025*	0.671
*N*	228	228	227	226	228	228	228	228	*228*	227
Serum insulin (mIU/L)										
Pearson Correlation	0.091	0.169^**∗**^	−0.068	0.001	0.109	0.026	0.090	−0.062	0.135^*∗*^	0.011
Sig. (2-tailed)	0.169	0.010	0.310	0.989	0.102	0.696	0.178	0.348	0.041	0.867
*N*	228	228	227	227	228	228	228	228	228	227
HOMA-IR^*∗∗∗*^										
Pearson Correlation	0.097	.290^*∗∗*^	−0.085	−0.035	0.125	−0.032	*0.143* ^*∗*^	*−0.143* ^*∗*^	*0.0322* ^*∗∗*^	0.001
Sig. (2-tailed)	0.146	0.000	0.199	0.598	0.060	0.627	*0.031*	*0.031*	*0.000*	0.989
*N*	228	228	227	227	228	228	*228*	*228*	*228*	227
HOMA % B^*∗∗∗∗*^										
Pearson Correlation	0.041	0.022	−0.029	0.022	0.041	0.042	0.021	0.014	−0.029	0.007
Sig. (2-tailed)	0.536	0.744	0.659	0.743	0.535	0.530	0.751	0.830	0.666	0.911
*N*	228	228	227	227	228	228	228	228	228	227
Urine albumin creatinine ratio (UACR)										
Pearson Correlation	0.107	0.114	−0.079	0.098	*0.154* ^*∗*^	0.083	*0.130*	*0.059*	0.049	*0.153* ^*∗*^
Sig. (2-tailed)	0.162	0.135	0.304	0.197	*0.042*	0.276	*0.088*	*0.443*	0.523	*0.044*
*N*	174	174	173	173	*174*	174	174	*174*	174	*173*
Systolic BP (SBP) mm of Hg										
Pearson Correlation	*0.125*	*0.143* ^*∗*^	0.112	0.020	0.078	−0.001	0.087	−0.048	*0.122*	−0.073
Sig. (2-tailed)	*0.058*	*0.029*	0.090	0.758	0.238	0.984	0.185	0.468	*0.065*	0.271
*N*	*232*	*232*	230	230	232	232	232	232	*232*	230
Diastolic BP (DBP) mm of Hg										
Pearson Correlation	*0.145* ^*∗*^	*0.160* ^*∗*^	0.056	0.006	0.110	0.031	0.096	−0.043	*0.130* ^*∗*^	−0.018
Sig. (2-tailed)	*0.028*	*0.015*	0.401	0.934	0.095	0.640	0.144	0.512	*0.047*	0.781
*N*	*232*	*232*	230	230	232	232	232	232	*232*	230

^**∗**^Correlation is significant at the 0.05 level (2-tailed); ^**∗∗**^correlation is significant at the 0.01 level (2-tailed); ^*∗∗∗*^Homeostasis Model Assessment for Insulin Resistance (HOMA-IR); ^*∗∗∗∗*^Homeostasis Model Assessment for insulin sensitivity (HOMA % B).

**Table 3:** Comparison of lipid indices among subjects with and without metabolic syndrome as per IDF criteria.

Lipid parameter	Metabolic syndrome (as per IDF criteria)	*N*	Mean	Std. dev	Sig. (2-tailed)^*∗∗*^
HDLc (mmol/L)	Present	121	0.94	0.25	*0.028*
	Not present	108	1.02	0.26	
mLDLc^*∗*^ (mmol/L)	Present	121	2.80	0.76	*0.013*
	Not present	107	2.56	0.66	
Non-HDLc (mmol/L)	Present	121	3.63	0.60	*0.002*
	Not present	108	3.36	0.65	
cLDLc^*∗∗*^ (mmol/L)	Present	121	2.79	0.52	0.569
	Not present	108	2.75	0.54	
sdLDLc^*∗∗∗*^ (mmol/L)	Present	121	0.92	0.33	*0.000*
	Not present	108	0.70	0.29	
lbLDLc (mmol/L)^*∗∗∗∗*^	Present	121	1.87	0.54	0.575
	Not present	108	1.83	0.51	
sdLDLc/lbLDLc	Present	121	0.55	0.51	*0.010*
	Not present	108	0.40	0.38	

^*∗*^Measured LDL-cholesterol (mLDLc); ^*∗∗*^calculated LDL-cholesterol (cLDLc) as per Friedewald's equation; ^*∗∗∗*^small dense LDL-cholesterol (sdLDLc); ^*∗∗∗∗*^large buoyant LDL-cholesterol (lbLDLc).

**Table 4:** Comparison of lipid indices among various groups formulated based upon the number of metabolic syndrome components present in subjects from 0 implying absence of any metabolic syndrome component to 5 implying all 5 components present in a subject.

Metabolic syndrome groups		Total cholesterol (mmol/L)	Fasting triglycerides (mmol/L)	HDLc (mmol/L)	mLDLc (mmol/L)	Non-HDLc (mmol/L)	cLDLc (mmol/L)	sdLDLc (mmol/L)^**∗∗**^	lbLDLc (mmol/L)^*∗∗∗*^	sdLDL/lbLDL	mLDLc/HDLc	VLDLc (mmol/L)
0	Mean	*4.33*	*1.07*	*1.15*	2.63	*3.18*	2.69	*0.67*	1.97	*0.34*	2.36	*0.21*
	*N*	*18*	*18*	*18*	18	*18*	18	*18*	18	*18*	18	*18*
	Std. dev	*0.49*	*0.31*	*0.18*	0.65	*0.53*	0.50	*0.24*	0.44	*0.08*	0.74	*0.06*

1	Mean	*4.31*	*1.28*	*0.99*	2.54	*3.31*	2.73	*0.65*	1.82	*0.37*	2.66	*0.26*
	*N*	*42*	*42*	*42*	41	*42*	42	*42*	42	*42*	42	*42*
	Std. dev	*0.69*	*0.73*	*0.27*	0.70	*0.68*	0.52	*0.33*	0.56	*0.53*	0.99	*0.15*

2	Mean	*4.43*	*1.54*	*0.97*	2.67	*3.45*	2.76	*0.80*	1.87	*0.45*	2.88	*0.31*
	*N*	*62*	*62*	*62*	62	*62*	62	*62*	62	*62*	62	*62*
	Std. dev	*0.63*	*0.62*	*0.26*	0.64	*0.62*	0.56	*.26*	0.48	*0.18*	0.87	*0.12*

3	Mean	*4.49*	*1.66*	*0.93*	2.77	*3.59*	2.80	*0.86*	1.90	*0.45*	3.03	*0.33*
	*N*	*46*	*46*	*46*	46	*46*	46	*46*	46	*46*	46	*46*
	Std. dev	*0.57*	*0.56*	*0.19*	0.80	*0.60*	0.54	*0.37*	0.50	*0.17*	0.88	*0.11*

4	Mean	*4.62*	*1.92*	*0.91*	2.79	*3.68*	2.84	*0.93*	1.86	*0.54*	3.16	*0.38*
	*N*	*36*	*36*	*36*	36	*36*	36	*36*	36	*36*	36	*36*
	Std. dev	*0.53*	*0.71*	*0.19*	0.82	*0.54*	0.45	*0.35*	0.57	*0.27*	1.02	*0.14*

5	Mean	*4.78*	*2.26*	*1.00*	2.68	*3.78*	2.76	*0.99*	1.69	*.8413*	2.9269	*0.45*
	*N*	*25*	*25*	*25*	25	*25*	25	*25*	25	*25*	25	*25*
	Std. dev	*0.56*	*0.99*	*0.39*	0.74	*0.69*	0.59	*0.29*	0.63	*1.03*	1.03	*0.19*

*p* value^**∗**^		*0.015*	*<0.001*	*0.001*	0.725	*0.001*	*0.896*	*0.000*	*0.910*	*<0.001*	0.055	*<0.001*

^*∗*^Kruskal Wallis Test. ^*∗∗*^Small density LDL-cholesterol (sdLDL-c) by Srisawasdi et al. regression equation. ^*∗∗∗*^Large buoyant LDL-cholesterol (lbLDL-c) by Srisawasdi et al. regression equation.

**Table 5:** Comparison of lipid indices among subjects with or without hypertension.

Lipid parameter	Hypertension	*N*	Mean rank	Asymp. sig.
Total cholesterol (mmol/L)	Absent	205	116.43	0.966
	Present	27	117.02	
Fasting triglycerides (mmol/L)	Absent	205	115.18	0.409
	Present	27	126.52	
HDLc (mmol/L)	Absent	203	115.47	0.985
	Present	27	115.72	
mLDLc (mmol/L)^*∗*^	Absent	204	115.98	0.760
	Present	26	111.75	
Non-HDLc (mmol/L)	Absent	205	116.85	0.825
	Present	27	113.81	
cLDLc (mmol/L)^*∗∗*^	Absent	205	118.01	0.345
	Present	27	105.04	
sdLDLc (mmol/L)^*∗∗∗*^	Absent	205	116.06	0.783
	Present	27	119.85	
lbLDLc (mmol/L)^*∗∗∗∗*^	Absent	205	117.85	0.399
	Present	27	106.26	
sdLDLc/lbLDLc	Absent	205	116.70	0.903
	Present	27	115.02	
mLDLc/HDLc	Absent	203	116.43	0.561
	Present	27	108.50	
VLDL-cholesterol (mmol/L)	Absent	205	115.18	0.409
	Present	27	126.52	

^*∗*^As per Friedewald's equation; ^*∗∗*^measured using nonparametric test (SPSS); ^**∗∗∗**^small density LDL-cholesterol (sdLDLc) by Srisawasdi et al. regression equation; ^**∗∗∗∗**^large buoyant LDL-cholesterol (lbLDLc) by Srisawasdi et al. regression equation.

**Table 6:** Comparison of lipid parameters among subjects with and without nephropathic changes as measured by urine albumin creatinine ratio (UACR).

Lipid parameter	Urine albumin creatinine ratio (UACR)	*N*	Mean	Std. deviation	Sig. (2-tailed)
Total cholesterol (mmol/L)	*<2.5 mg/g*	135	4.36	0.54	0.006
	*>2.4 mg/g*	39	4.6	0.45	
Fasting triglycerides (mmol/L)	*<2.5 mg/g*	135	1.47	0.669	0.082
	*>2.4 mg/g*	39	1.68	0.63	
HDLc (mmol/L)	*<2.5 mg/g*	134	.9951	0.27	0.084
	*>2.4 mg/g*	39	.9254	0.20	
mLDLc (mmol/L)^*∗*^	*<2.5 mg/g*	134	2.6166	0.73662	0.413
	*>2.4 mg/g*	39	2.7156	.63762	
Non-HDLc (mmol/L)	*<2.5 mg/g*	135	3.3656	.59011	*0.000*
	*>2.4 mg/g*	39	3.6797	.43173	
cLDLc (mmol/L)^*∗∗*^	*<2.5 mg/g*	135	2.6997	.52434	*0.007*
	*>2.4 mg/g*	39	2.9110	.38825	
sdLDLc (mmol/L)^*∗∗∗*^	*<2.5 mg/g*	135	.7586	.35181	0.172
	*>2.4 mg/g*	39	.8315	.27045	
lbLDLc (mmol/L)^*∗∗∗∗*^	*<2.5 mg/g*	135	0.4277	.32988	0.411
	*>2.4 mg/g*	39	0.4599	.16809	
sdLDLc/lbLDLc	*<2.5 mg/g*	134	2.7685	.88169	0.103
	*>2.4 mg/g*	39	3.0651	1.01300	
mLDLc/HDLc	*<2.5 mg/g*	135	.2950	.13228	0.082
	*>2.4 mg/g*	39	.3360	.12625	

^*∗*^As per Friedewald's equation; ^*∗∗*^measured using independent sample *t*-test (SPSS); ^**∗∗∗**^small density LDL-cholesterol (sdLDLc) by Srisawasdi et al. regression equation; ^**∗∗∗∗**^large buoyant LDL-cholesterol (lbLDLc) by Srisawasdi et al. regression equation.
